# Malodour classification with low-cost flexible electronics

**DOI:** 10.1038/s41467-023-36104-z

**Published:** 2023-02-11

**Authors:** Emre Ozer, Jedrzej Kufel, John Biggs, Anjit Rana, Francisco J. Rodriguez, Thomas Lee-Clark, Antony Sou, Catherine Ramsdale, Scott White, Suresh Kumar Garlapati, Palaniappan Valliappan, Aiman Rahmanudin, Venuskrishnan Komanduri, Glenn Sunley Saez, Sankara Gollu, Gavin Brown, Piotr Dudek, Krishna C. Persaud, Michael L. Turner, Stephanie Murray, Susan Bates, Robert Treloar, Brian Newby, Jane Ford

**Affiliations:** 1 Pragmatic Semiconductor, 400 Cambridge Science Park, Milton Road, Cambridge, CB4 0WH UK; 2 Arm, 110 Fulbourn Rd, Cambridge, CB1 9NJ UK; 3 Indian Institute of Technology Hyderabad, Department of Materials Science and Metallurgical Engineering, Indian Institute of Technology Hyderabad, Hyderabad, 502285 India; 4 University of Manchester, University of Manchester, Oxford Road, Manchester, M13 9PL UK; 5 Unilever, Port Sunlight Lab, Quarry Road East, Bebington, Wirral CH63 3JW UK

**Keywords:** Electrical and electronic engineering, Chemical engineering

## Abstract

Understanding body malodour in a measurable manner is essential for developing personal care products. Body malodour is the result of bodily secretion of a highly complex mixture of volatile organic compounds. Current body malodour measurement methods are manual, time consuming and costly, requiring an expert panel of assessors to assign a malodour score to each human test subject. This article proposes a technology-based solution to automate this task by developing a custom-designed malodour score classification system comprising an electronic nose sensor array, a sensor readout interface and a machine learning hardware fabricated on low-cost flexible substrates. The proposed flexible integrated smart system is to augment the expert panel by acting like a panel assessor but could ultimately replace the panel to reduce the test and measurement costs. We demonstrate that it can classify malodour scores as good as or even better than half of the assessors on the expert panel.

## Abstract (web summary)

Designing machine learning hardware on flexible substrates is promising for several applications. Here, the authors propose an integrated smart system built with low-cost flexible electronics components for classifying human malodour, and demonstrates that the proposed system scores malodour as good as expert human assessors.

## Introduction

Skin, the largest organ in human body, is covered with glands that secrete body odour. Body odour is complex and composed of a variety of volatile organic compounds (VOCs). Odour produced by sweat secreted from underarms has more VOCs than the odour in urine or saliva, some of which are distinct for individuals which can be used for biometric fingerprinting and disease diagnosis^1^. Also, underarm odour has cues of diet, sex, age, hygiene, health and reproductive status^2^.

Prior studies in the literature^3–8^ focus on identifying key VOCs in sweat and detecting them with the help of electronic nose (e-nose) sensors and electronics. Typically, gas sensors are used to collect data and then digitised by a data acquisition electronic system. The digital data is, then, analysed by a computer running one of the known classification algorithms. The common characteristic of these studies is that they rely on expensive and bulky gas sensors for detecting VOCs, and conventional electronics for data analysis and classification. Lorwongtragool et al.^9^ proposed printed carbon nanotubes and other polymer composite-based e-nose sensor array to classify armpit odour, and relied on conventional electronic equipment to couple with the printed e-nose sensor array.

The global deodorant business will reach a market size of $30Bn by 2026^10^, and the key to maintaining a business of this size is the ability to design and test capability at speed, be cost effective and be globally relevant. Understanding malodour is a key driver for personal care companies as the link between consumer perception and malodour content is essential to measure the benefit of new product technologies.

New methods to measure, sample and analyse malodour that are comparable to current physical measurements in use are highly desirable. Analysis of samples without direct human contact has become increasingly important with the desire to build understanding in new areas and between different groups of people and conditions. Character profiling combined with data from odour sensing analysis can drive learnings and speed in innovation and understanding in combatting body malodour. Previous work with sensors and e-nose type assessments^11–15^ have limitations which are difficult to overcome in terms of sampling and measurement from the armpit. The environment is warm, humid and the compounds of interest are highly volatile and easily lost to the atmosphere during sampling.

The current sampling method in use is to use a panel of individuals that are undergoing assessment and a physical sniff assessment of the underarms by a panel of expert assessors. The assessors rate the armpit for odour and assign a score rating which is on a nonlinear numeric scale. This score is then used to determine the given test result whether that be testing of product efficacy or otherwise. Typically, this score is the mean of the expert assessors individual scores and is assigned to each underarm. This is termed a mean malodour score or MMS. In this article, the MMS scale ranges from 0 to 4–0 being no malodour (or blank) and 4 being strongest malodour. These test methods are physical, time consuming and costly, and ‘in the field’ assessment is a challenge due to the complex sample matrix and sampling methods required to capture and retain highly volatile molecules.

There is a need to develop a smart sensor system that can compete with the current manual test methods in capacity, sensitivity, and accuracy in a cost-effective manner. The comparable perception test of the underarms and scale rating is the desired outcome of the smart sensor system. The measurement comparison between sensor reading and underarm malodour is the key requirement and unlocking this would increase the opportunity for in-field testing to be undertaken or testing at speed to further increase our understanding of body odour.

Flexible electronics allow electronic components developed on substrates such as plastic or paper, and offers thinness, conformability, and low manufacturing costs over conventional silicon-based electronics. Flexible components such as sensors, organic displays, printed batteries, analogue interface, and processors can be assembled to build flexible integrated smart systems^16^.

This article describes the methodology of analysing human armpit malodour to drive the development of a low-cost flexible integrated smart system consisting of organic FET (OFET)-based e-nose sensors, metal-oxide thin film transistor (TFT)-based sensor readout interface (SRI) and machine learning engine (MLE) for malodour classification. E-nose sensor arrays are custom designed and fabricated on a polyethylene naphthalate (PEN) substrate whilst the SRI and MLE are custom designed, integrated and fabricated as a flexible integrated circuit or FlexIC on a polyimide substrate. Armpit malodour detected by the flexible OFET sensor array and digitised by the flexible metal-oxide TFT-based SRI is then classified by the flexible metal-oxide TFT-based MLE.

The malodour classification performance of the proposed flexible integrated smart system is quantified using malodour fabric swatches, validated, and demonstrated against a conventional Si-based microcontroller. The experimental results show that it can classify the armpit malodour score as good as or even better than half of the human armpit sniffing panel.

We envisage that the flexible integrated system will be a single-use ‘smart swatch’ that can substitute ordinary fabric swatches used today to sample armpit malodour of a subject. A subject can rub the sensor part of the smart swatch underarm and the FlexIC will predict the malodour score and display it on the smart swatch, and the entire smart swatch is then disposed for hygiene reasons. The focus of the article is to integrate all three components (i.e., gas sensors, SRI, and MLE) into one system and to demonstrate that inexpensive and small form-factor plastic sensor and electronic components can be put together to solve an important problem in the deodorant industry. The malodour classifying flexible integrated smart system architecture developed in this article can be adapted to other odour prediction applications such as food freshness, air quality, odour nuisance management, and odour-based biometrics.

## Results

An experimental framework is set up to collect sensor data using fabric swatches from custom-developed e-nose sensor devices. Test subjects, from whom fabric swatches are collected, must be male and female participants of varying ages. The reason for this is as there are known differences in the odour of males and females at a perception level. To differentiate between males and females allows a more personalised approach for the physiological difference between males and females within a deodorant product. The details about the fabric swatch analysis and data collection methodology are described in “Methods”.

**Fig. 1:**
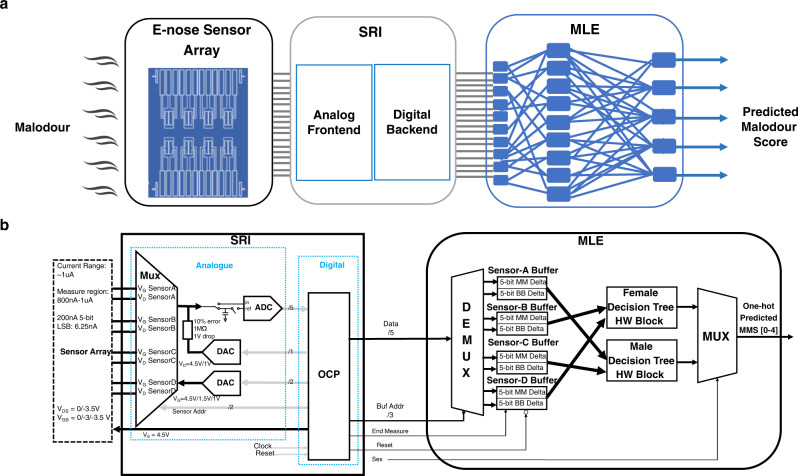
Flexible integrated smart system. **a** The high-level system architecture for armpit malodour classification problem. **b** Hardware blocks of the SRI and MLE used in the integrated FlexIC are interfaced to the sensor array.

The high-level architecture of the flexible integrated smart system developed in the article is shown in Fig. 1a. The e-nose sensor array consisting of different OFETs is exposed to the sensor array. The current output of the individual OFET devices is pre-processed by the analogue frontend of the SRI, converting it into a voltage, and then digital values by an analogue-to-digital converter (ADC). In the final step, the digitised values are fed into the MLE to classify the malodour into one of the pre-determined malodour scores.

**Fig. 2:**
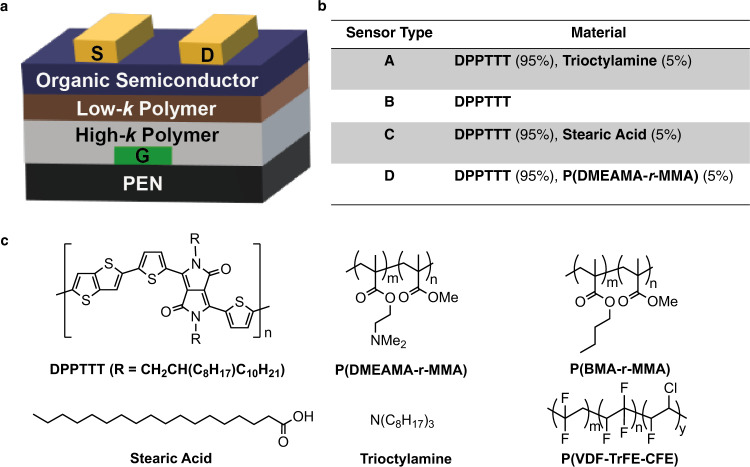
OFET devices as VOC sensors. **a** Schematic of bottom gate, top contact OFET used in this work (S, D, and G are source, drain, and gate electrodes, respectively). **b** Compositions of the organic semiconductor layers used in the four different types of OFET (A–D). **c** Structures of all of the materials used in the OFET devices—low-*k* polymer is cross-linked P(BMA-r-MMA) and high-*k* polymer is P(VDF-TrFE-CFE).

The OFET devices of the sensor array act as cross-reactive VOC sensors. The array contains devices made using four different types of semiconductors (A, B, C, and D) that respond to different components of the VOCs in the malodour fabric swatches. Figure 2 shows the cross-section of an OFET device, four sensor material types and their notations.

The OFETs are based on a bottom-gate top-contact transistor configuration with an all-polymer bilayer gate dielectric (see Fig. 2a)^17^. Two important design considerations for fabrication of a low-voltage OFET chemical sensor are that: (i) the organic semiconductor (OSC) layer has to be placed in a position that permits exposure to the analyte, i.e., in a bottom-gate device geometry, and (ii) the gate dielectric material requires a large areal capacitance and good electrical insulation, but it needs to be chemically robust to withstand the solvents used to solution process the OSC layer in this geometry. The bilayer dielectric is composed of a high dielectric constant relaxor ferroelectric fluoropolymer, P(VDF-TrFE-CFE), to enable low-voltage operation, that is coated with a thin layer of a low dielectric constant, photo-crosslinked, methacrylate-based copolymer, P(BMA-co-MMA) that interfaces with the organic semiconductor leading to minimal hysteresis, representative transfer characteristics for the four types of OFETs and collated extracted performance parameters are available in “Methods”. The active OSC is based on a copolymer of a substituted diketopyrrolopyrrolethiophene and thienothiophene (DPPTTT). This polymeric semiconductor was blended with a range of additives (trioctylamine, stearic acid or a random copolymer of an amino substituted acrylate and methylmethacrylate, P(DMAEMA-r-MMA), to help differentiate the headspace of the malodour fabric swatches. The structures and compositions of the materials used are shown in Fig. 2b, c.

**Fig. 3:**
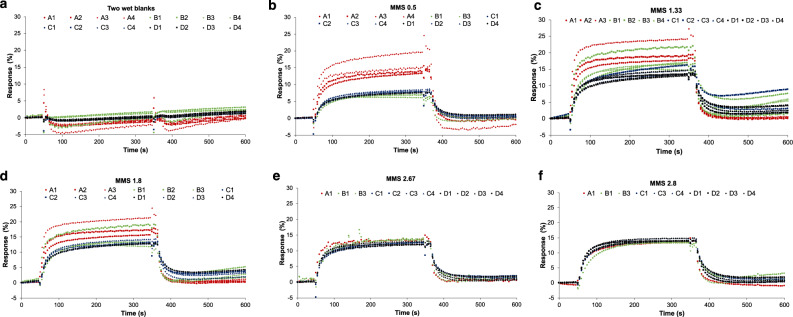
Sensor responses to various fabric swatches. **a** Wet blank swatch fabrics. **b**–**f** Malodour swatch fabrics from different MMS values. A–D refer to four different types of OFET devices, and multiple sensors of the same type are used in the experiments.

The fabricated OFET sensors show very low response to wet-blank fabric swatches (see Fig. 3a) but show larger responses when exposed to malodour fabric swatches of different MMS values (see Fig. 3b–f). The magnitude of the responses is generally higher for higher MMS-value fabric swatches for sensors C and D, which reflects the observation that the concentration of VOCs in the headspace of these fabrics is, in general, proportional to MMS but the response of sensors A and B is larger at low values of MMS and decreases at higher MMS values.

**Fig. 4:**
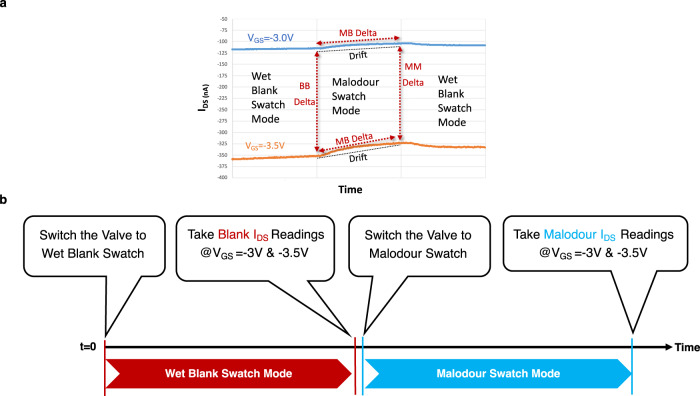
Operation modes and measurement protocol. **a** Delta measurements to mitigate the sensor drift issue across different *V*_GS_ voltage and swatch modes. MB Delta refers to the *I*_DS_ difference between the malodour (M) swatch and blank (B) swatch modes at the same *V*_GS_. BB Delta and MM Delta refer to the *I*_DS_ difference at the two *V*_GS_ voltages in the same mode. **b** The basic measurement protocol is shown for collecting the sensor outputs in the experiments where the wet blank swatch is used as a reference. First, the sensors are exposed to wet blank swatch and two readings of *I*_DS_ at two *V*_GS_ voltages (i.e., −3 and −3.5 V) are recorded. Then the sensors are exposed to the malodour swatch and two readings of *I*_DS_ at the same two *V*_GS_ voltages are recorded.

To minimise the effects of sensor drift during the measurements, the sensor current outputs are read at two different voltages at *V*_GS_ = −3V and *V*_GS_ = −3.5 V. The difference in measured current (Δ*I*_DS_) at these two voltages are recorded for both wet blank (i.e., no malodour) and malodour swatches as shown in Fig. 4a. Δ*I*_DS_ measurements are taken between the two *V*_GS_ voltages at the same mode as well as between the different modes at the same *V*_GS_. *I*_DS_ readings from the sensor array when the sensor is exposed to swatches are denoted by B for wet blank and M for malodour swatches. Figure 4b describes the basic measurement protocol used to collect the sensor output data for wet blank and malodour swatches at two *V*_GS_ voltages.

The sensor array was fabricated and used to collect malodour datasets in order to drive the software-based design space exploration (DSE) framework for exploring machine learning (ML) models as well as the evaluation of the system demonstrator platforms. The best ML model fitting the datasets is identified in the DSE framework, and then implemented as an MLE. The DSE explores a pool of the known ML model space, various Δ*I*_DS_-based features, sensor types, and different sensor data quantisation levels. The details about the DSE framework can be found in “Methods”.

A new metric called “Goodness of Prediction” is proposed to quantify how good is the predicted score with respect to the individual scores given by the panellists given the true MMS. Although the predictor is designed to predict an MMS value, what is important is to quantify how good is the predicted score with respect to the panellists who assign their malodour scores leading to the true MMS. The “Goodness of Prediction” is calculated as follows:

- Measure the distance between the predicted MMS and true MMS
- Measure the distance between the individual panellist score and true MMS
- If the distance in (a) is equal to or less than the distance in (b), the prediction is as good as or better than the panellist.

The DSE is performed separately for male and female datasets to find the best configurations per sex, and the results are presented in Table 1. The highest-performing (in terms of prediction performance) configurations for both sexes are shown in the first and fourth rows of the table. The highest-performing configuration for female datasets achieves 73% average goodness of prediction, which uses 7-bit quantised sensor data, three sensors, a feature of “MB Delta@*V*_GS_ = −3.5 V” and the Decision Tree as the ML algorithm. Data quantisation level is the key parameter for assessing the MLE hardware design complexity. Although a lower data quantisation level has a negative impact on the prediction performance, it improves the hardware area and power consumption in the ADC where the selected data quantisation level determines the ADC resolution. The complexity of a flash ADC depends on the number of bits as an n-bit flash ADC needs 2^n^ -1 analogue comparators. For example, the number of analogue comparators can go up to 127 in a 7-bit ADC from 31 in a 5-bit ADC. For this reason, we also show the best 6-bit and 5-bit data quantisation configurations for both sexes. The best 6-bit and 5-bit data quantisation configurations also use the Decision Tree. The best 6-bit data quantisation configuration sacrifices 1 percentage point in performance save 64 analogue comparators in the ADC whilst the best 5-bit data quantisation configuration can reduce the number of analogue comparators by 96 at the cost of 7 percentage points loss in performance. Thus, we select the best 5-bit configuration as the configuration of the MLE hardware design for female swatches because it allows a simpler ADC in the SRI, which uses less chip area and consumes less power. This configuration uses sensors A & B, the features extracted from each sensor called “MM Delta & BB Delta”, each feature having 5 bits, and the Decision Tree as the ML model. The details of the feature extraction and nomenclature can be found in “Methods”.

**Table 1:** Average goodness of prediction results

Gender	Configuration	Sensor combination	Feature	Quantisation level	ML algorithm	Average goodness of prediction (%)
Female	Highest-performing	A, C & D	MB Delta@ *V*_GS_ = −3.5 V	7 bits	Decision Tree	73
Female	Best 6-bit	A & B	Delta of Delta	6 bits	Decision Tree	72
Female	Best 5-bit	A & B	MM Delta & BB Delta	5 bits	Decision Tree	66
Male	Highest-performing	A & C	MM Delta & BB Delta	7 bits	Support Vector Machine	73
Male	Best 6-bit	A & C	MM Delta & BB Delta	6 bits	Decision Tree	72
Male	Best 5-bit	A & C	MM Delta & BB Delta	5 bits	Decision Tree	70

Each malodour swatch has sex and malodour scores given by the individual panellists, and the average of the scores determining the true MMS value for the experiment. At the end of each experiment, the current outputs of the four sensors for both blank and malodour swatches are stored along with the true MMS value. The total number of female and male datasets (or fabric swatches) is 64 and 115, respectively. A 5-run cross-validation is used to avoid overfitting when we run each configuration, and therefore an average of the 5 runs is presented as “Average Goodness of Prediction”. The first three rows of the table show the results for female swatches whilst the last three rows show the results for male swatches. See “Methods” for the description of the features.

The highest-performing configuration for male datasets as shown in the fourth row of Table 1 also achieves 73% average goodness of prediction, which also uses 7-bit quantised sensor data but a Support Vector Machine as the ML algorithm. Similar to the female datasets, the best 6-bit and 5-bit data quantisation configurations for male datasets are also shown in the table. The best 6-bit and 5-bit data quantisation configurations have 1 and 3 percentage points less prediction performance with respect to the highest-performing configuration that has a 7-bit data quantisation level. By the same argument made for female subjects, the best 5-bit data quantisation configuration saves more chip area and power consumption in the SRI due to a simpler ADC design. In summary, the best 5-bit configuration is selected to design the MLE hardware for male swatches. This configuration uses sensors A & C, the feature set “MM Delta & BB Delta”, 5 bits per feature and the Decision Tree.

The integrated FlexIC, as shown in Fig. 1b, consists of the SRI and MLE blocks. The SRI is made up of two sub-blocks: digital and analogue. The digital block, called the Order Code Processor (OCP), is based on a custom-designed simple instruction set, and controls all the OFET sensors, the analogue block, and interfaces with the MLE. The analogue block is responsible for controlling the sensor measurement protocol as well as for digitising the sensor outputs using a 5-bit ADC.

**Fig. 5:**
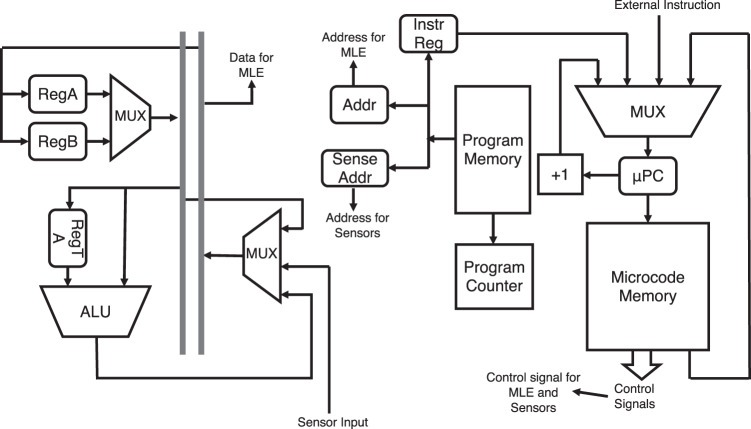
Order Code Processor (OCP). The components in OCP (digital SRI block) are shown. OCP supports 16 different instructions. There are three registers each of which has a 5-bit width. The fixed code sits in the programme memory where each instruction is microcoded in the microcode memory. The programme and microcode memory sizes are 256 and 832 bits, respectively.

The architecture for the OCP is shown in Fig. 5. The OCP is designed to be generic and easily reconfigurable with a set of core functions already provided. There are two types of instructions: 1-word instruction with 4-bit code and 2-word instruction with 8-bit code. The set of instructions themselves is stored in the programme memory that can be easily changed to modify the SRI behaviour. The programme memory is used to include all the required instructions, while the microcode memory generates the control signals to execute each instruction in the programme memory.

The analogue block consists of the analogue multiplexer (MUX), a 5-bit flash ADC and two digital-to-analogue (DAC) converters. The analogue MUX is a 4:1 data selector device which connects the gate and drain terminals of one of the four sensors to the ADC/DAC for control and measurement of the sensors.

**Fig. 6:**
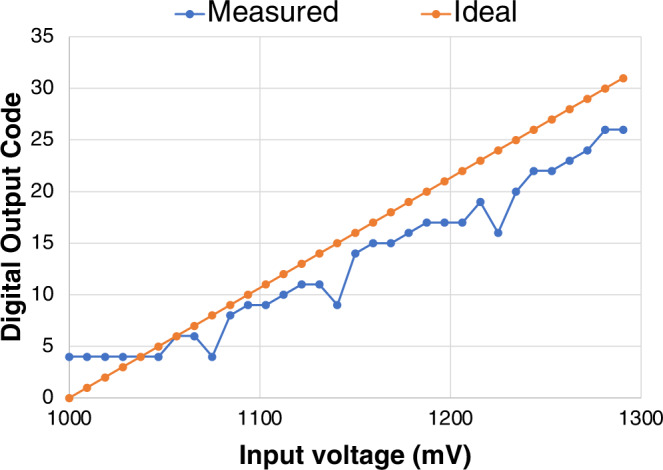
ADC measurement. The measured ADC output is shown in 300 mV range at 1C offset with respect to the ideal ADC output.

The analogue multiplexer connects the gate and drain terminals of each sensor selected by the sensor address sent from the OCP to the necessary *V*_G_ and *V*_D_ voltages generated by the DAC that uses a resistor ladder. *V*_S_ is always at 4.5 V. Then, the sensor’s drain current is converted to a voltage by a resistor. There are two measurements from each sensor—one at *V*_GS_ = −3V and another at −3.5 V. An internal capacitor is charged from the first measurement at *V*_GS_ = −3V, which is applied to the ADC as a reference voltage, and this charge is subtracted from the second measurement at *V*_GS_ = −3.5 V, which is applied as the input to the ADC. The resulting value (i.e., a delta voltage between the input and reference voltages) representing the “MM Delta” or “BB Delta” feature is sent to the ADC. A voltage drop across the resistor causes an error in the voltage applied to the ADC for conversion. An input range of 300 nA across a 1 MΩ resistor results in an expected 300 mV voltage range at the input to the 5-bit ADC (see Fig. 6).

The OCP is programmed to condition the sensors for 5 min and then take measurements from the sensors after the end of this period. Measurement cycles are performed for both wet blank and malodour swatches, data is assigned an address value and sent to the MLE for processing.

**Fig. 7:**
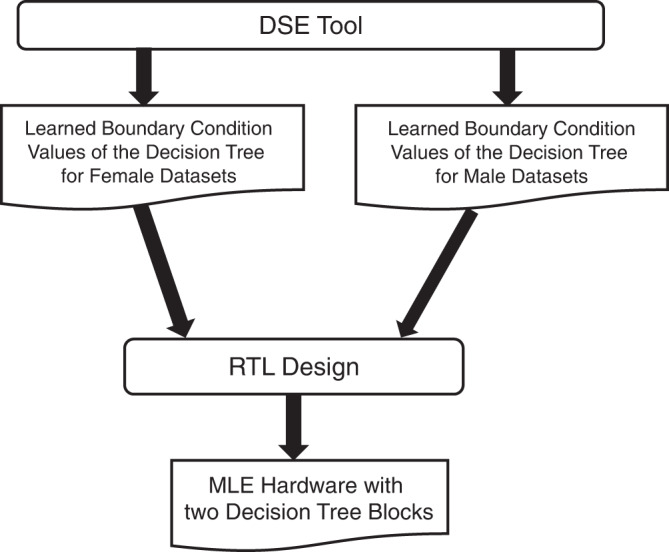
MLE hardware generation flow. The stages for creating MLE hardware from the design space exploration (DSE) tool are shown.

The MLE hardware is designed using the parameters finalised during the DSE framework. Figure 7 shows the flow from the DSE to the MLE hardware design. The MLE contains two decision tree hardware blocks—one for each sex, and one of the decision tree hardware blocks for a given sex input is selected to classify the malodour. The DSE tool generates the learned boundary conditions and structure of each decision tree after the training stage. The decision trees are then translated into hardware description by RTL design. A decision tree consists of a series of if-then-else statements. An if-then-else statement is implemented as a comparator and a multiplexor logic in hardware. A comparator compares sensor value to a boundary condition value that is learned during the ML training. Each branch of if-then-else statements terminate at a predicted MMS value.

The inputs to the MLE hardware are the 5-bit Delta data, 3-bit sensor buffer address, a 1-bit end measurement, a 1-bit sex select, and a reset from SRI. The output from the hardware is a one-hot encoded 5-bit predicted MMS data directly connected to the LEDs. SRI sends each 5-bit delta data in a serial mode along with the 3-bit sensor address. SRI triggers the end measurement signal after sending eight 5-bit delta values. Once MLE receives the end measurement signal, all eight values in the sensor buffers are fed into the decision tree hardware blocks. The sex select input selects the predicted MMS of the associated sex. The details about the decision tree structure, learned boundary values, and conditional statements can be found in “Methods”.

**Fig. 8:**
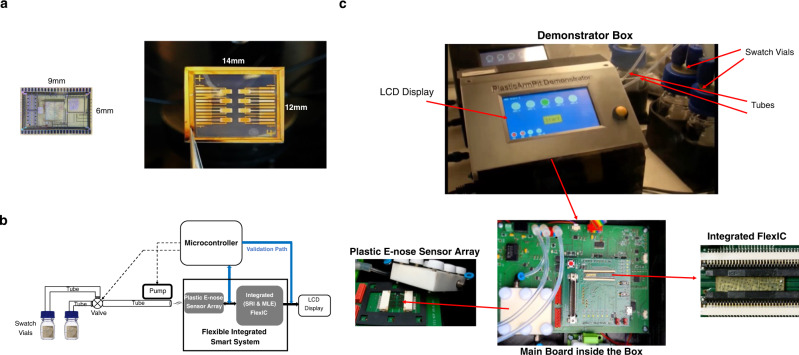
System prototypes. **a** Die photos of the FlexIC integrating SRI and MLE (left), and OFET devices fabricated on a PEN substrate (right). The sensor array has eight sensors made up of four types of OFETs with redundant replicas. Only four sensors are used and powered on. **b** The high-level architecture of the demonstrator system. The blank and malodour swatches are placed into two vials. The headspace in vials is sucked into the tubes through the pump. The vial selection is controlled by a valve that allows only the headspace from the selected vial to be sucked into the main tube. The long tube carries the headspace onto the plastic e-nose sensor array. The sensor outputs are captured both by the microcontroller on the validation path and the FlexIC on the flexible integrated smart system. The FlexIC takes the sensor outputs and predicts its malodour score. The validation path uses the same e-nose sensor array but emulates the functions of the SRI and MLE in software and runs them on the microcontroller. This implies that the ADC in the microcontroller is used to digitise the sensor outputs from the e-nose sensor array but scaled back to 5 bits. The predictions from both systems are displayed on the LCD screen. **c** Picture of the demonstrator box—the valve and pump are not shown in the pictures, and the microcontroller is located on the backside of the main board. The swatch vials are not part of the box and are connected through the tubes to the box.

Figure 8a shows the die photos of the FlexIC integrating the SRI and MLE blocks (left), and the e-nose sensor array (right). The FlexIC is fabricated using a commercial ‘fab-in-a-box’ manufacturing line, FlexLogIC®^18^ using an n-type 0.8 µm indium-gallium-zinc-oxide (IGZO) TFT technology on polyimide. More details about the fabrication process and methodology can be found in our earlier works^19, 20^. The FlexIC occupies an area of 9 mm by 6 mm. The total NAND2-gate equivalent gate count of the digital blocks (Digital SRI + MLE) is around 2100. The integrated chip is powered with a supply voltage of 5 V, consumes 10.2 mA, and operates at 8 kHz. The chip has 44 pins (top and bottom) for interconnecting the sensor arrays, external display, power and ground, and additional test probe pins (left and right). The e-nose sensor array has two replicas of 4 OFET devices on PEN with an area of 12 mm × 14 mm, and dissipates only 14 µW of power.

Figure 8b shows how the full system where the mechanical equipment and electronics are integrated to validate the armpit classification concept. Figure 8c shows the demonstrator box and its internal hardware. A demonstrator box is designed to accommodate all the electronics, including the main board, FlexIC, plastic e-nose sensor array, a microcontroller, an LCD display, a valve, and a pump. More details of the system integration and validation can be found in “Methods”.

The malodour score predictions from both the flexible integrated smart system and the validation path (i.e., microcontroller + e-nose sensor array) and their predictions are measured and tabulated in Table 2. The flexible integrated smart system approach achieves an average goodness of prediction of 50% for both female and male swatches by predicting malodour scores as good as or better than the half of the panellists. On the other hand, the validation path predicts the malodour scores more accurately (i.e., 83% for female and 71% for male swatches) than the flexible integrated smart system. This is mainly because the ADC in FlexIC is inaccurate in converting sensor values to correct digital outputs, which, at times, may impact the predictions made by the MLE.

**Table 2:** The performance results of the two system prototypes

System	Average goodness of prediction for female swatches	Average goodness of prediction for male swatches
Flexible Integrated Smart System	50%	50%
Validation Path (E-nose SensorArray + Microcontroller emulating the integrated SRI and MLE in Software)	83%	71%

The first system is the flexible integrated smart system, and the second system is the microcontroller-based validation system used as a reference. A total of 51 malodour swatches (27 female and 24 male) were tested on the system. The choice of these swatches was made at random to ensure no bias is allowed.

## Discussion

In this article, we have shown that a low-cost flexible integrated smart system consisting of an e-nose sensor array made of OFETs on a flexible substrate coupled with a flexible integrated circuit can be a reliable tool for industries that rely on human panels for odour quality assessment. This can eliminate the health hazards encountered by human panels in sniffing such samples and transform the way odour quality is assessed—not only for malodour but for food quality, cosmetics, forensic, medical diagnostics, and others.

There are large differences in composition between malodour emitted from female as opposed to male test subjects, and so panellists normally assess these samples separately. A notable achievement has been the sensitisation of OFETs to the malodourous compounds of interest in sweat samples, and predicting the malodour scores using low-cost electronics achieving performance as good or better than half of the panellists for both female and male test subjects.

We have demonstrated an interdisciplinary achievement of combining materials, sensors, flexible IC fabrication, and software developments incorporating our understanding of how humans assess malodour scores to achieve a workable low-cost system.

## Methods

The participants of the study wear a t-shirt, and a fabric swatch is worn in the underarm of the t-shirt, and this becomes the sample that undergoes analysis using the measurement methodology described herein.

### Measurement

The measurement setup to sample the headspace above a fabric swatch is shown in Supplementary Fig. 1. It consisted of charcoal filters, Duran glass bottles for the fabric swatches (100 ml volume), a 3-way valve, 3-way connectors, sensor boards with sensors and a pump to suck the air at a defined flow rate. The measurement setup was placed in an incubator at a constant temperature that was set to 32 °C. The glass bottles with Duran caps have inlet and outlet tubes through which air can be sucked to sample the headspace from either a wet blank swatch (clean swatch with 0.5 ml added water) or a malodour fabric swatch. Before commencing any measurement, the bottles containing the wet blank and the malodour fabric swatches were kept closed for 60 min to create a consistent headspace of humid air. For the baseline measurement, air was drawn through the bottle containing the wet blank fabric swatch, by opening the 3-way valve, and the headspace was passed over the sensors in boards 1 and 2. After measuring the baseline of humid air from the blank swatch for 5 minutes the three-way valve was switched to sample the headspace of the bottle containing the malodour fabric swatch for 5 min by drawing the air through the bottle. To complete the measurement cycle, the 3-way valve was switched back to the wet blank fabric bottle to recover the sensors to the baseline.

### OFET devices

The OFET devices were fabricated as described previously^17^. Representative transfer characteristics for the four types of devices and collated extracted performance metrics are collated in Supplementary Fig. 2.

### Design space exploration (DSE) for ML model development

Supplementary Fig. 3 shows the DSE tool that crawls in the design space to search for the best configuration in terms of the sensor combination, a feature, an ML model, and the quantisation level of sensor data.

There are four sensor types leading to 24 different sensor combinations. The focus is to create Δ*I*_DS_-based features derived from the sensor output values to eliminate sensor drift issue. Supplementary Table 1 shows six different Δ*I*_DS_-based features and their descriptions.

Data quantisation level determines the hardware complexity. For example, the complexity of the SRI and MLE will be much smaller if the data bit-width is 4 bits rather than 8 bits. The data bit-width determines the size of the ADC in the SRI as well as the logic in the SRI digital or OCP and MLE. Thus, multiple data quantisation levels from 3 bits to 7 bits are explored in the DSE framework.

Finally, five standard ML models (Support Vector Machine, K-Nearest Neighbour, Multi-layer Perceptron, Gaussian Naïve Bayes and Decision Tree) are used. Typical ML workflow is used in the experiments, e.g., a dataset (male or female) is split into two training and test datasets, and ML models are trained offline using the training dataset, and the performance of the trained models are evaluated using the test dataset. A 5-run cross-validation is used to avoid overfitting, and we use “average goodness of prediction” over these five runs.

### Decision tree structures

The MLE hardware consists of two decision trees one for each sex, and their structures are shown in Supplementary Figs. 4 and  5. Each decision tree consists of many if-then-else blocks and has a depth of 9 and 6 for female and male datasets, respectively. Each condition in the if-then-else block is learned during the training phase, and is constant during the inference, in which case it is a hardwired value in the hardware implementation.

### Details in system integration and validation

Both digital SRI (i.e., OCP) and MLE blocks were validated separately in the FlexIC using the additional test pins provided on the IC as shown in Supplementary Fig. 6. For each block, input–output test vectors were generated from the datasets. The input test vectors were applied to the testports of a block, and the output were monitored. Then, the test outputs were compared to the expected output vector data. For digital SRI, the input test vectors were driven to the SRI testport at the top, and the analogue block is bypassed through a multiplexer. The output of the digital SRI is captured through another SRI port at the bottom. Similarly, the test input vectors were fed through the MLE testport at the top and the digital SRI were bypassed through the two multiplexers and the output is captured on the output pins of the FlexIC on the right (shown in red). In this test setup, each block passed all the test vectors (i.e., the outputs of all test vectors matched the expected outputs—achieving 100% test accuracy). The MLE block alone can operate as fast as 160 kHz.

A data acquisition system was developed as shown in Supplementary Fig. 7 that allowed routing of four OFET gas sensors to the inputs of the FlexIC or alternatively to a conventional circuit that measured OFET currents, digitised these, and presented the signals to a software MLE based on a decision tree. The FlexIC was mounted on a printed circuit board (PCB) containing a microcontroller that allowed handshaking and routing of signals to and from the main board.

The measurement of OFET currents with the FlexIC involved placing a resistor between source and ground of the OFET and measuring the voltage across this resistor when the current between the source and drain of the transistor changed in response to an odour. In this case, the OFET was placed in the saturated regime with a fixed *V*_DS_, and with *V*_GS_ being switched between −3 and −3.5 V.

For the main data acquisition system, a multiplexed current to voltage converter together with user programmable *V*_DS_ and *V*_GS_. On this board were eight OFET gas sensors organised in pairs of different sensor types for redundancy. One of each pair of sensors is routed to the FlexIC on receipt of a suitable handshake signal. The FlexIC processes the signals and routes the output of the MLE encoded as 0–4 back to the main board that displays the output on a screen and transmits data to an external computer via USB.

The main board also controlled switching between a control and sample via a three-way solenoid valve, as well as a small pump that aspirated air from the sample bottles across the sensor array. The microcontroller board would send a logic line low to take control from the main board, initialising the routing of sensor signals to the FlexIC. The main board responds with a signal going from low to high. The control sample is measured, and a logic pulse high is sent that triggers the main board to switch to the sample of interest. After measurement of that is completed and results are available, the FlexIC handshake signal is sent high and the main board responds by sending its handshake line high, reads the results, and displays it.

ZIF connectors were used to interface the FlexIC to its PCB. The connectors provided a non-permanent connection that was low resistance and robust. This simplified the assembly process for the novel FlexIC design, allowing shorter assembly-test cycle times compared with permanent attach of the FlexIC via conductive paste or similar. Before assembly, the FlexICs underwent on-wafer testing, and the wafers were then diced and released following Pragmatic’s standard wafer processing. The validated FlexIC’s were picked manually from wafer frame using tweezers and inserted into the opposing ZIF connectors. The flexible nature of the FlexIC allowed it to be manipulated into the static connectors. Due to the thinness of the FlexIC compared with the FPCs (Flexible Printed Circuits) that the ZIF connectors are designed for, a 9 × 6 mm thicknesser made from 240 μm thick clear acetate sheet was inserted above the FlexIC to make up the difference and enable clamping of the FlexIC within the connector. The installed FlexIC was tested post assembly using the same test scheme as used to validate the FlexIC on wafer, with the test equipment interfaced to the FlexIC PCB via a VHDCI cable. This test validated the connections between FlexIC and PCB.

## Supplementary information

Supplementary Information

## Data Availability

The data used in tables and figures are available from the corresponding author upon request.

## Code availability

The code of the decision tree algorithms is available from the corresponding author upon request.
