# Supplementary material for: Malodour classification with low-cost flexible electronics
Source: Nat Commun. 2023 Feb 11;14:777. doi: 10.1038/s41467-023-36104-z (PMC9922322; doi:10.1038/s41467-023-36104-z)
Supplement: Supplementary file 1 — Supplementary Information [file 41467_2023_36104_MOESM1_ESM.pdf]

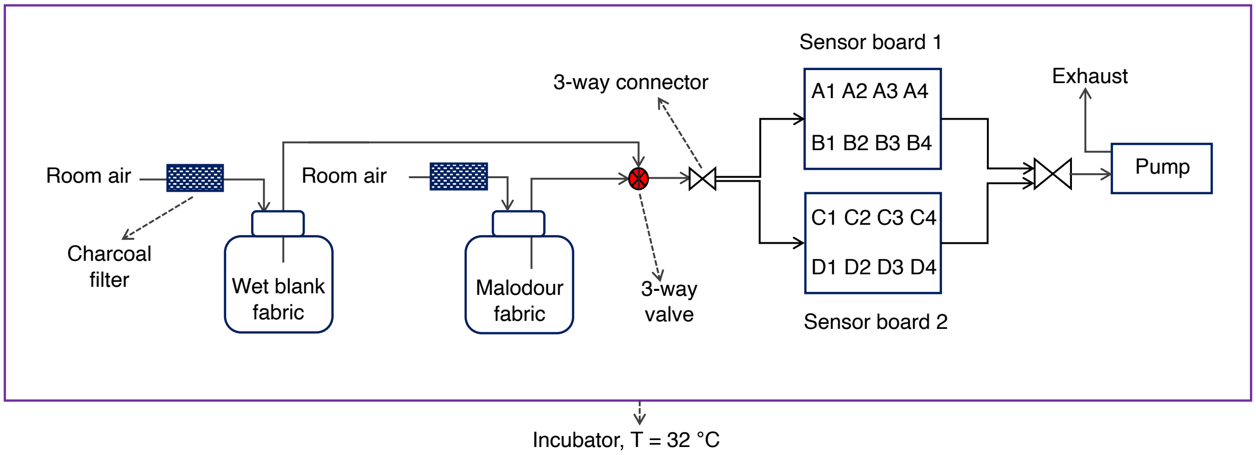

Supplementary Fig. 1 Swatch measurement setup

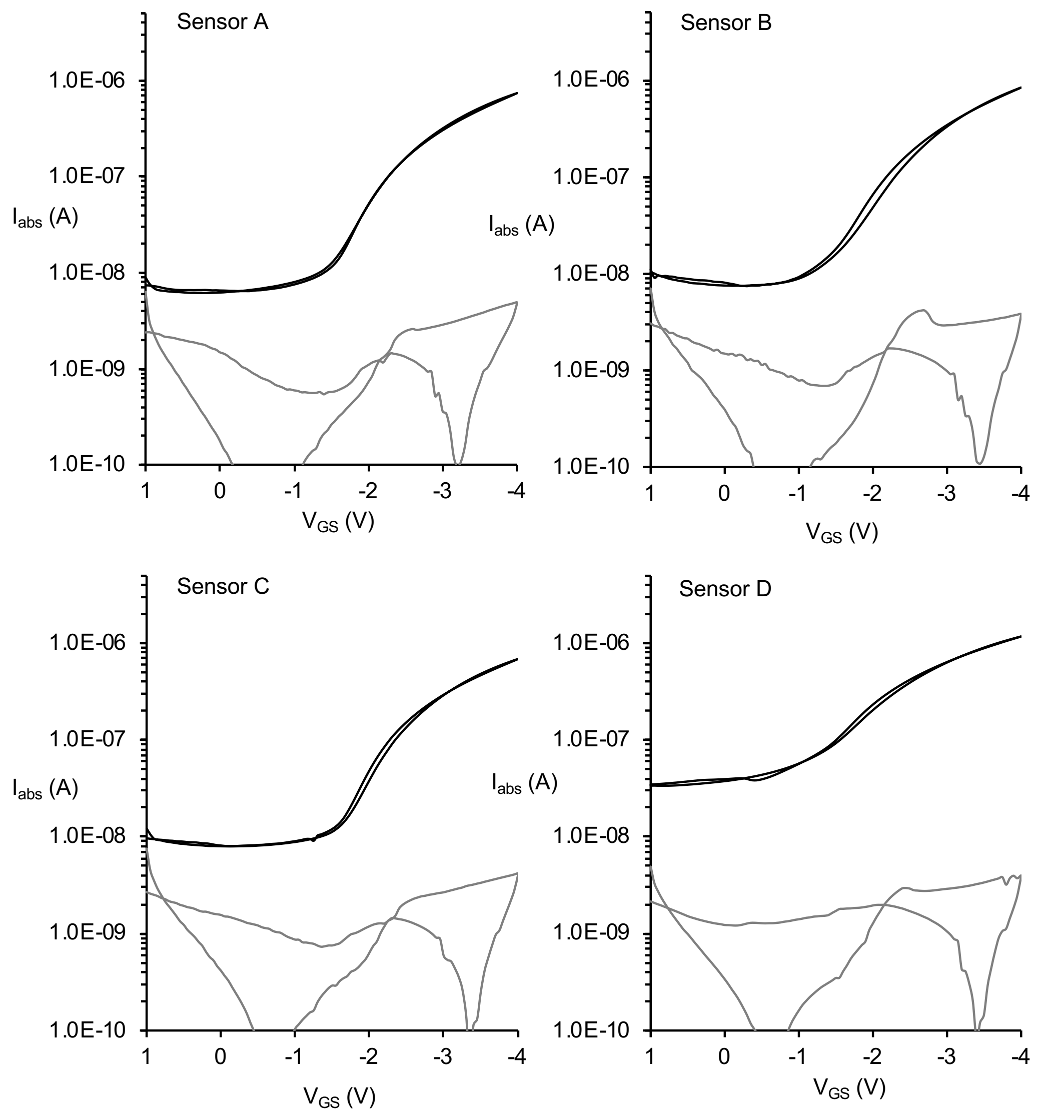

Metric	Sensor A	Sensor B	Sensor C	Sensor D
V_{th} (V)	-0.8 +/-0.5	-1.6 +/-0.02	-1.6 +/-0.2	-0.7 +/-0.3
μ (cm^2/Vs)	0.088 +/-0.01	0.085 +/-0.008	0.071 +/-0.01	0.070 +/-0.003

Supplementary Fig. 2 OFET sensor device characterization. Double sweep transfer curves for all four OFET sensors from V_{GS} = 1 to −4 V under constant V_{DS} = −4 V are shown. Extracted performance metrics for these OFETs (W = 2 mm, L = 60 mm, C_i = 82 nF/cm^2) are shown below the figures from a minimum of 18 working devices fabricated on 3 separate substrates.

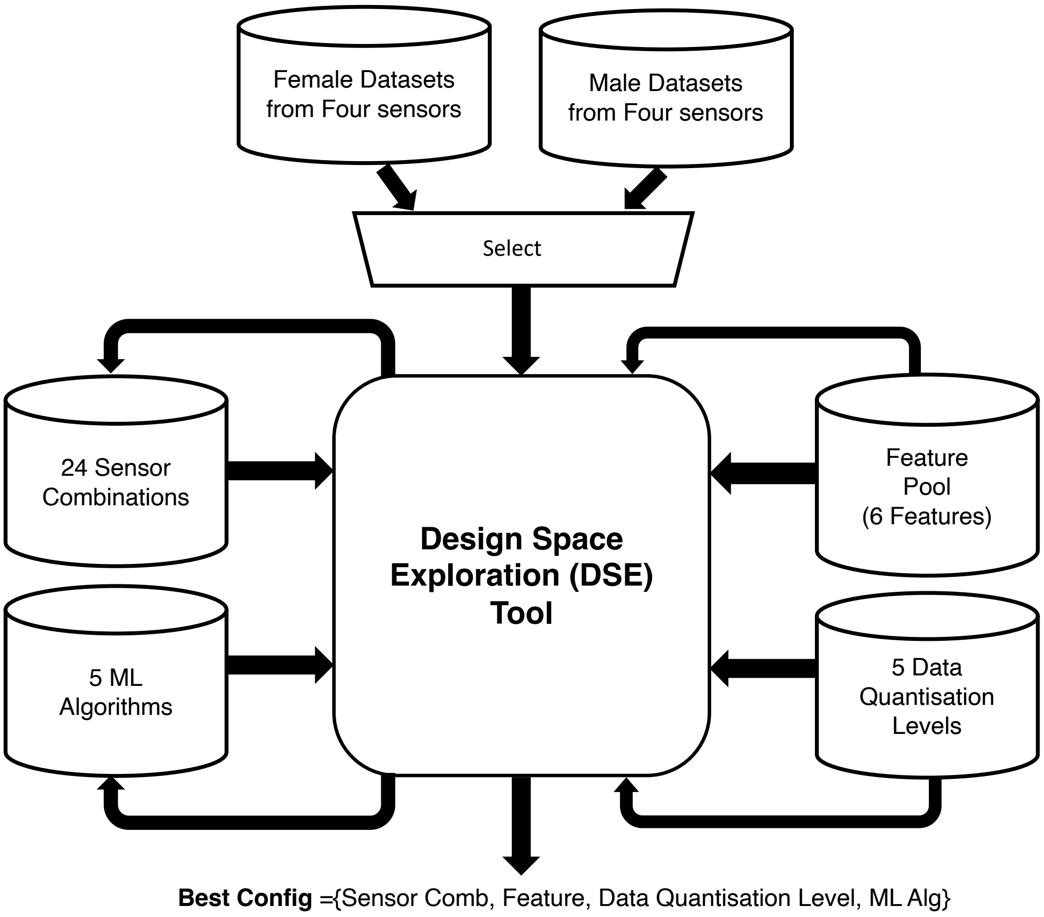

Supplementary Fig. 3 Design space exploration (DSE) tool. The DSE tool has four input parameters: Feature, quantisation level, ML algorithm and sensor combination. The tool explores a space of 3,600 different design configurations. The exploration is performed for male and female datasets separately. The output is the best configuration of a specific sensor combination, feature, data quantisation level and an ML algorithm.

Feature	Description
MB Delta@V_{GS} = -3V	Difference between M and B values for all sensors at V_{GS} = -3V
MB Delta@V_{GS}= -3.5V	Difference between M and B values for all sensors at V_{GS} = -3.5V
MB Delta@both V_{GS}'es	Differences between M and B values for all sensors at both V_{GS} = -3V & V_{GS} = -3.5V
MM Delta	Difference between M value at V_{GS} = -3.5V and M at V_{GS} = -3V for all sensors
MM Delta & BB Delta	Difference between M value at V_{GS} = -3.5V and M at V_{GS} = -3V for all sensors<br>&<br>Difference between B value at V_{GS} = -3.5V and B at V_{GS} = -3V for all sensors
Delta of Delta	(MM Delta – BB Delta) values for all sensors

Supplementary Table 1 Six different delta-based features used in the design space exploration – B: Sensor output value for the wet Blank swatch @VGS and M: Sensor output value for the Malodour swatch @VGS.

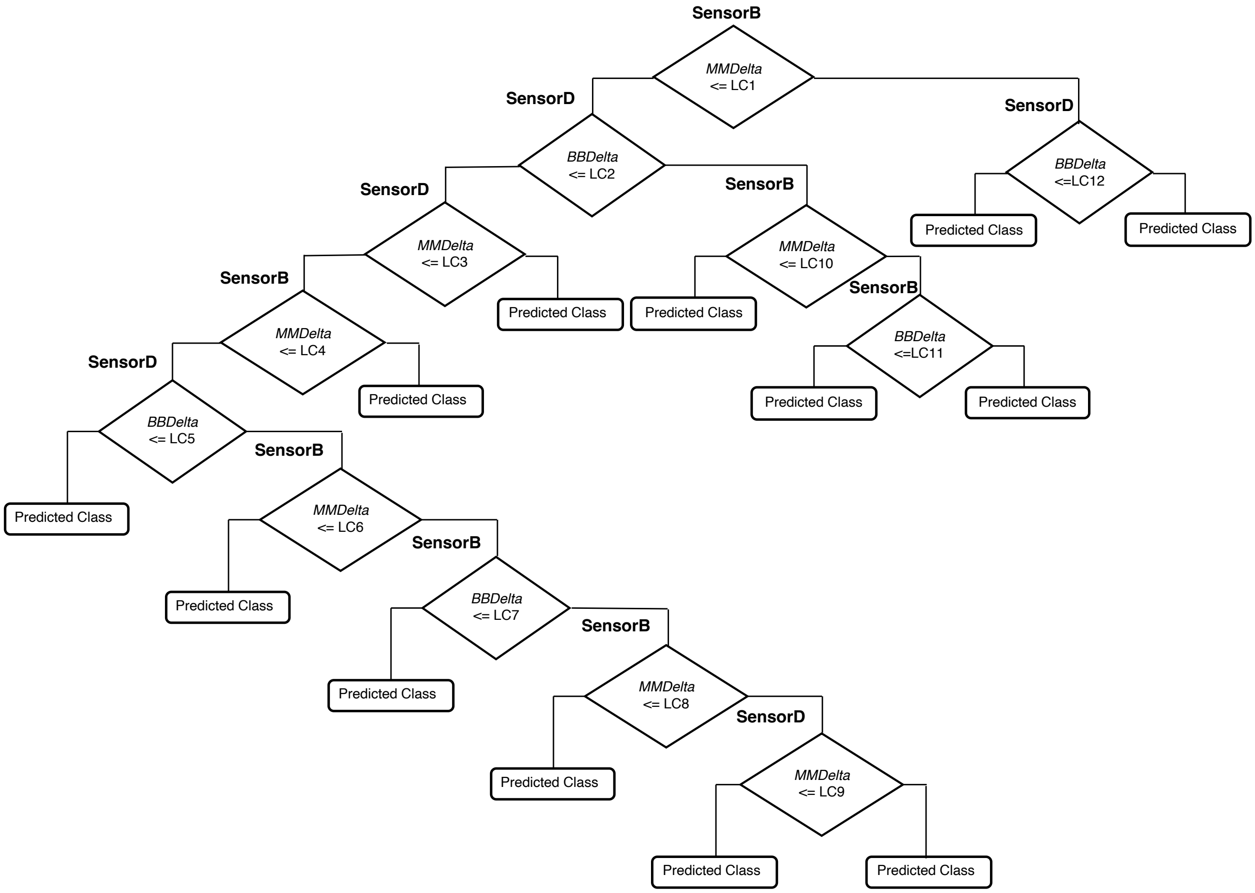

Supplementary Fig. 4 The decision trees for female datasets. BBDelta and MMDelta are the features, and LC refers to learned constants. Branching ends in a predicted class value.

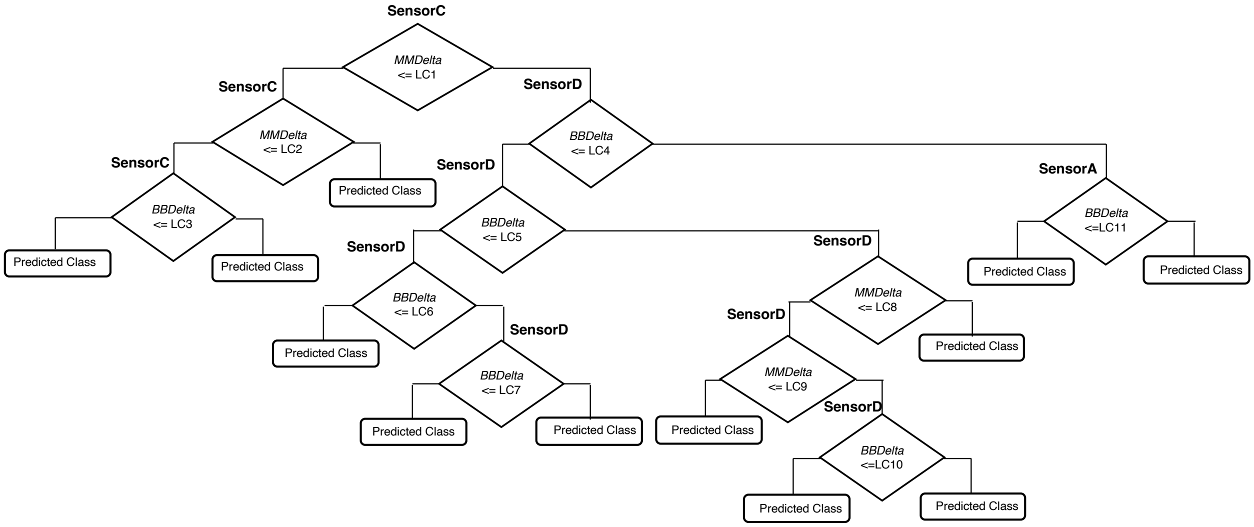

Supplementary Fig. 5 The decision trees for male datasets. BBDelta and MMDelta are the features, and LC refers to learned constants. Branching ends in a predicted class value.

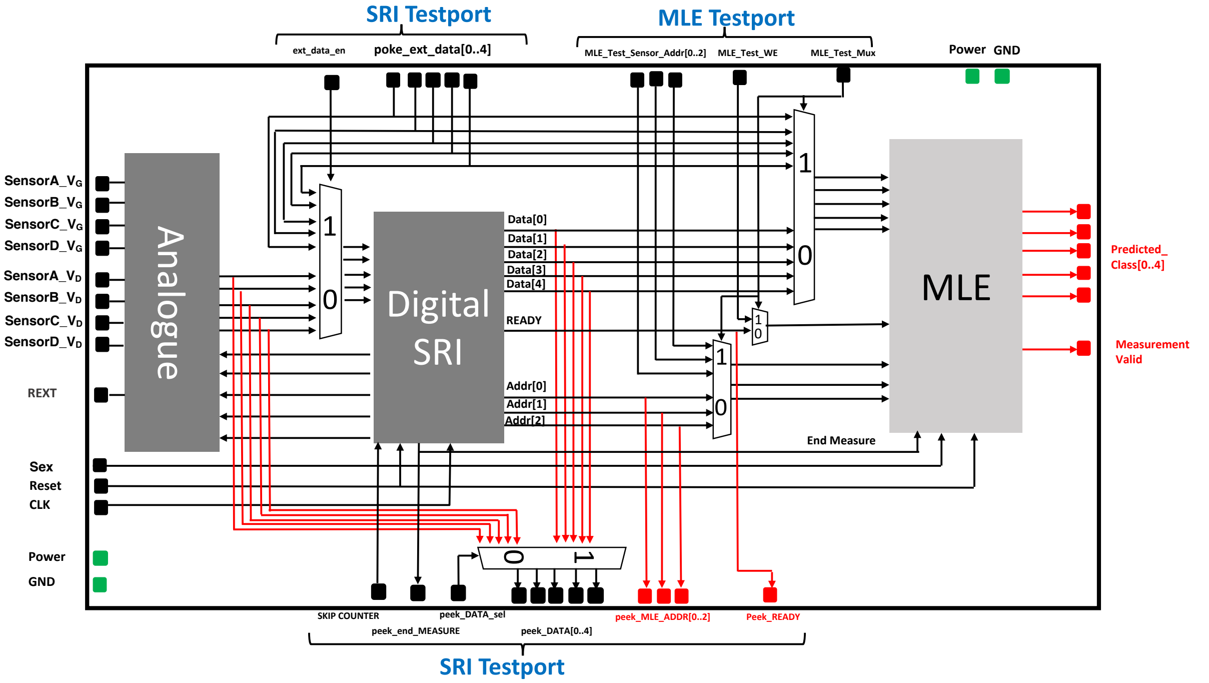

Supplementary Fig. 6 Testing individual blocks. Additional test ports are added to test individual blocks inside the integrated FlexIC.

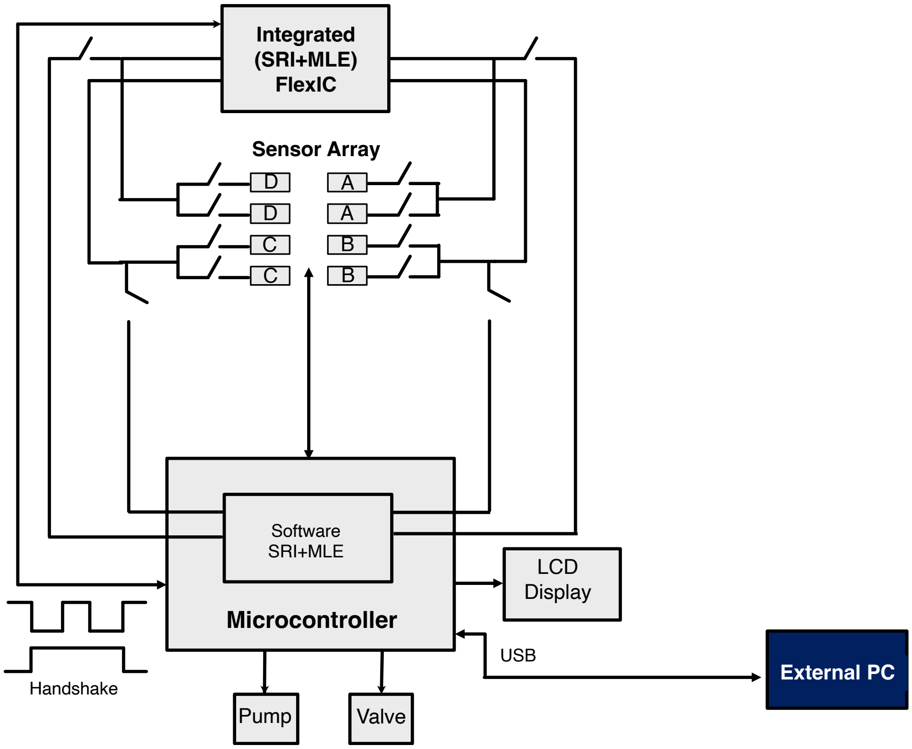

Supplementary Fig. 7 Main data acquisition system
